# Teneurin Structures Are Composed of Ancient Bacterial Protein Domains

**DOI:** 10.3389/fnins.2019.00183

**Published:** 2019-03-13

**Authors:** Verity A. Jackson, Jason N. Busby, Bert J. C. Janssen, J. Shaun Lott, Elena Seiradake

**Affiliations:** ^1^MRC Laboratory of Molecular Biology, Cambridge, United Kingdom; ^2^School of Biological Sciences, The University of Auckland, Auckland, New Zealand; ^3^Crystal and Structural Chemistry, Bijvoet Center for Biomolecular Research, Faculty of Science, Utrecht University, Utrecht, Netherlands; ^4^Department of Biochemistry, University of Oxford, Oxford, United Kingdom

**Keywords:** cell adhesion, teneurin, bacterial toxin, evolution, choanoflagellate

## Abstract

Pioneering bioinformatic analysis using sequence data revealed that teneurins evolved from bacterial tyrosine-aspartate (YD)-repeat protein precursors. Here, we discuss how structures of the C-terminal domain of teneurins, determined using *X*-ray crystallography and electron microscopy, support the earlier findings on the proteins’ ancestry. This chapter describes the structure of the teneurin scaffold with reference to a large family of teneurin-like proteins that are widespread in modern prokaryotes. The central scaffold of modern eukaryotic teneurins is decorated by additional domains typically found in bacteria, which are re-purposed in eukaryotes to generate highly multifunctional receptors. We discuss how alternative splicing contributed to further diversifying teneurin structure and thereby function. This chapter traces the evolution of teneurins from a structural point of view and presents the state-of-the-art of how teneurin function is encoded by its specific structural features.

## Introduction

Teneurins are evolutionarily ancient cell surface receptors which have emerged as important regulators of many neurobiological processes in both vertebrates and invertebrates, including synaptic partner matching (Hong et al., 2012), synaptic organization (Mosca et al., 2012), neuronal migration (Drabikowski et al., 2005) and axonal guidance and targeting (Leamey et al., 2007; Berns et al., 2018). This variety of functions has been attributed to both homophilic interactions between teneurins expressed on adjacent cells and also heterophilic interactions with other cell surface receptors, such as the synaptic adhesion G-protein-coupled receptor, latrophilin, which is essential for hippocampal synapse formation in mice (Silva et al., 2011; Boucard et al., 2014; Anderson et al., 2017). The relative importance of heterophilic vs. homophilic interactions of teneurins is not fully understood.

**FIGURE 1:**
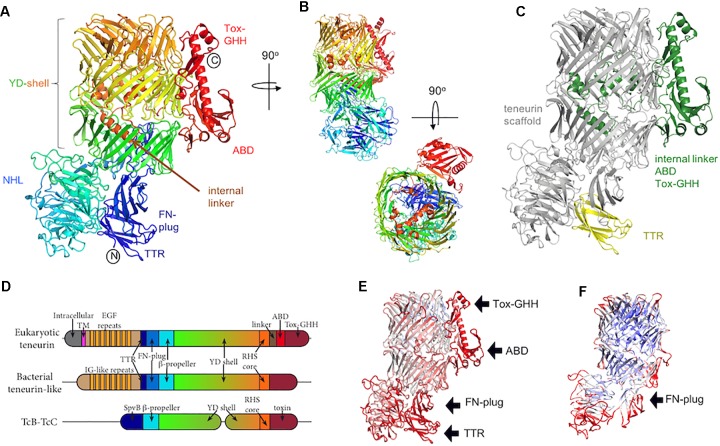
The teneurin C-terminal region. **(A)** The crystal structure of chicken Ten2 (PDB 6FB3) is colored according to the rainbow (blue, N-terminus; red, C-terminus). The position of each subdomain is indicated. **(B)** Alternative views of the chicken Ten2 crystal structure with the NHL domain facing (upper) and a top view (lower). **(C)** Chicken Ten2 is shown with the teneurin scaffold in gray, upstream transthyretin-like (TTR) domain in yellow, and downstream linker, antibiotic-binding-like domain (ABD) and Tox-GHH domains in green. **(D)** Schematic of the subdomains found in vertebrate teneurins, a bacterial teneurin-like protein from *Bacillus subtilis* (WP_088111228.1), and TcB/TcC toxins. **(E)** The model of chicken Ten2 (chain D) is colored according to the temperature factor (B-factors), from low (min = 30 Å^2^, blue) to high (max = 150 Å^2^, red). The black arrows point out the least rigidly ordered areas of the crystal structure, which have the highest B-factors. **(F)** The Cryo-EM model of murine Ten3 (PDB 6FAY) is shown, colored according to resolution, from low (max = 4.6 Å, red) to high (min = 3.7 Å, blue). The TTR, ABD and Tox-GHH domains are missing from this model.

Consistent with their evolutionarily ancient origins, bioinformatic and phylogenetic analyses have revealed that eukaryotic teneurins arose via a horizontal gene transfer event from bacteria early during metazoan evolution (Tucker et al., 2012). However, their relationship to these bacterial proteins has remained unclear due to a lack of structural data for the teneurins. The structural analysis of teneurins has long been hampered by difficulties with recombinant expression and purification of these large, glycosylated and intricately folded type II transmembrane protein receptors. The advent of advanced eukaryotic expression systems, especially those using mammalian HEK293 cells (Seiradake et al., 2015), and insect cells in suspension (Berger and Poterszman, 2015) have made the production of high quality samples tractable and led to the recent publications of the first teneurin structures (Jackson et al., 2018; Li et al., 2018). These recently published structures agree with each other overall, but there are some discrepancies that cannot be easily explained by differences in the sequences used, and which may arise from the methods employed to determine the structures. Where there are differences, we focus on the results obtained with the most completely modeled and highest resolution structure (solved by *X*-ray crystallography, up to 2.4 Å resolution), which is that of chicken Teneurin 2 (Figure 1A,B; Jackson et al., 2018). This structure is in good agreement with the structure of murine Teneurin 3, solved by cryo-electron microscopy by the same authors (up to 3.8 Å resolution), and with the structure of human Teneurin 2 solved by Li et al. (2018), also using cryo-electron microscopy (up to 3.1 Å resolution). The structure of chicken Teneurin 2 (residues 955-2802) obtained by X-ray crystallography is >99% complete, although less structural rigidity is observed in its peripheral domains. This is reflected by increased thermal motion (B-factors) in these regions (Figure 1E). The two models derived from cryo-electron microscopy reconstructions are both less complete compared to the *X*-ray structure, in particular in those regions corresponding to those with the highest B-factors in the crystal structure (Figure 1F). This suggests that these areas of the protein may be inherently flexible, but what causes the differences in magnitude of the domain flexibility in the different structures is currently not clear. Given the availability of the higher resolution and more complete *X*-ray structure, we will only discuss the lower resolution cryo-EM structures where the data is most reliable.

Alongside their cryoEM structure of human Teneurin2, Li et al. (2018) also present compelling mutagenesis and functional data. To avoid overlap with other chapters of this review series we focus our discussion on the structural features of teneurins with respect to their evolution from the bacterial protein ancestors.

## The Teneurin Scaffold

Teneurins are typically ∼2800 amino acids long with about half of this sequence encoding a structurally conserved extracellular scaffold (Figure 1B). Over half of this scaffold region contains a sequence repeat motif known as a tyrosine-aspartate (YD)-repeat [or rearrangement hotspot (Rhs)-repeat] (Zhang et al., 2012). The YD-repeat motif is widespread amongst prokaryotic proteins, and has been structurally characterized in the context of the heterodimeric TcB-TcC subcomplex in bacterial ABC toxin complexes (Busby et al., 2013; Gatsogiannis et al., 2013; Meusch et al., 2014). In both teneurins and TcB-TcC complexes, each YD-repeat forms a pair of β-strands joined by a β-hairpin. Together, multiple repeats form an extended spiraling β-sheet resulting in a large, hollow shell-like structure (the YD-shell) that is sealed at the C-terminal end by spiraling inward to form an “Rhs-associated core domain”-like structure. As well as acting as a central scaffold, the teneurin YD-shell is thought to bind to negatively charged heparin glycans (Minet et al., 1999). A mutation in the YD-shell of human Ten1, P1610L, is linked to congenital anosmia (Alkelai et al., 2016).

Two smaller domains are found N-terminal to the YD-shell and complete the teneurin scaffold. The most N-terminal of these domains is a distinctive fibronectin (FN) type-III domain termed the FN-plug domain (Figure 1A,D). Compared to a canonical FN type-III domain, the FN-plug contains an insertion (44 amino acids in chicken Ten2) between β strands 1 and 2 and an extension of its C-terminus (38 amino acids in chicken Ten2). This insertion and extension form a separate subdomain designated the “plug,” as it resides inside the YD-shell cavity, forming numerous hydrophobic and hydrogen bonding interactions with the shell interior. The YD-shell and FN-plug combination encloses a space of ∼130 nm^3^.

Between the FN-plug and YD-shell is a six-bladed β-propeller domain (Figure 1A,B,D), the NHL domain. The NHL domain has been shown to determine the specificity of teneurin homophilic interactions (Beckmann et al., 2013), which are thought to underlie initial synaptic partner matching in the murine hippocampus (Berns et al., 2018), the vertebrate visual system (Dharmaratne et al., 2012; Antinucci et al., 2013) and in the *Drosophila* olfactory map (Hong et al., 2012) and neuromuscular junction (Mosca et al., 2012). The NHL domain is positioned perpendicular to the YD-shell and is held in place by the FN-plug domain. The top face (Chen et al., 2011) of the NHL domain is decorated with extended loops, stabilized by five highly conserved disulphide bonds. One of these loops undergoes alternative splicing. Recent *in vitro* data indicates that inclusion of this splice site in Ten3 enables homophilic interactions between teneurins expressed on adjacent cells “*in trans*” (Berns et al., 2018). Consistent with these residues being important for homophilic teneurin interactions *in trans*, teneurin isoforms including this alternatively spliced region have shown cell–cell adhesive properties (e.g., for Ten2 Beckmann et al., 2013), whilst an isoform lacking these residues did not show any adhesion (Silva et al., 2011; Boucard et al., 2014). Recent reports have also indicated that inclusion of this splice site also abrogates the human teneurin-latrophilin interaction (Li et al., 2018), however, the alternatively spliced chicken Ten2 retains its binding capabilities (Jackson et al., 2018). Thus this area requires further investigation to provide meaningful mechanistic insights across species.

## Bacterial Teneurin-Like Proteins

### Comparison of Teneurin and TcB-TcC

The C-terminal teneurin scaffold shows a strong similarity to the TcB-TcC subcomplex of bacterial ABC insecticidal toxin complexes (Tc), with some significant differences. These ABC toxins are large protein complexes produced by a number of bacterial species against insect targets. These toxin systems are comprised of three major components: TcA forms a pentameric injection apparatus that binds to an insect cell and punctures the cell membrane, injecting the toxic component (Landsberg et al., 2011; Gatsogiannis et al., 2013). Together TcB and TcC form a continuous hollow shell-like structure that contains the cytotoxic cargo (the C-terminal end of TcC) until delivery (Busby et al., 2013; Meusch et al., 2014). We will compare the C-terminal domain of Teneurin with the combined TcB-TcC complex, as the latter heterodimer acts as a single functional unit. The fact that teneurins are produced as a single large polypeptide, whereas TcB-TcC is a heterodimer of two separate proteins (TcB and TcC), is likely the result of a genetic rearrangement, as both TcB and TcC are required to form a properly folded complex, and the C-terminus of the TcB protein is located directly adjacent to the N-terminus of TcC (Busby et al., 2013; Meusch et al., 2014). The *tcB* and *tcC* genes can be fused to produce a single protein that folds correctly (Busby and Lott, unpublished data), and this type of fusion can be found naturally in certain bacterial species such as the TcdB2 protein from *Burkholderia pseudomallei*.

**FIGURE 2:**
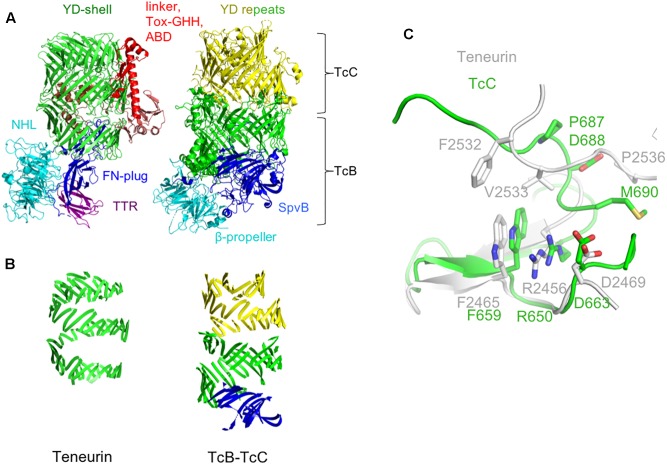
Comparison of the teneurin scaffold and peripheral domains with the TcB-TcC complex. **(A)** Chicken Ten2 (left) is shown in the same orientation as the TcB-TcC complex (right), with structurally equivalent regions colored accordingly (green and yellow: YD repeats, cyan: NHL domain and β-propeller, dark blue: FN-plug and SpvB domain). **(B)** The YD-repeat regions of both proteins, illustrating the larger size of the shell in TcB-TcC. **(C)** Structural alignment of the teneurin (gray) and TcC (green) Rhs-associated core-like domain at the autoproteolytic cleavage site of TcC.

Overall, the TcB-TcC shell is larger than the equivalent region of teneurin (2177 for the TcB + TcC2-NTD complex from *Yersinia entomophaga* vs. 1458 amino acids for chicken teneurin 2). This results in the spiral of β-sheet making ∼1 extra turn in TcB-TcC compared to teneurin (Figure 2A,B). This difference suggests that the internal cavity formed by the YD-shell can vary in size by simply having a different number of YD-repeats, potentially allowing differently sized protein cargo to be encapsulated. The “cargo” proteins encapsulated by TcB-TcC complexes are typically in the range of 276–295 amino acids. The linker region that is retained inside Ten2 is only 90 amino acids, although the teneurin FN-plug domain also extends into the central cavity.

Where teneurin has a TTR and FN-plug domain upstream of the NHL β-propeller, TcB proteins have an SpvB “domain” that extends the YD-shell. In teneurin these domains cause the β-propeller to be folded out to one side, with the FN-plug domain sealing the opening to the shell’s internal cavity. However, in TcB proteins this opening is plugged by the β-propeller and SpvB domain. In both cases, the exterior face of the β-propeller is involved in protein-protein interactions: in teneurin, this surface is involved in homophilic interactions (Beckmann et al., 2013), whereas in TcB this is the interaction surface that attaches to the pentameric TcA toxin delivery device. In both teneurins and TcB, the β-propeller forms a separate domain to the YD-shell.

Aside from the N-terminal TTR and FN-plug domains, the basic arrangement of teneurin and TcB-TcC is fundamentally similar. In both structures the β-propeller domain is followed by a series of YD-repeats that form a spiral of β-sheet around a central cavity, capped at the C-terminal end by the Rhs-repeat-associated core domain. This domain serves to both seal the hollow “shell” at the C-terminal end and, in TcC, acts as a self-cleaving aspartic protease to cut loose the “cargo” protein (Busby et al., 2013; Meusch et al., 2014). While there are a number of residues in this domain that are conserved between teneurins and TcC proteins (chicken Ten2 and the TcC protein YenC2 are 26.4% identical and 32.2% similar in amino acid sequence), the highly conserved DxxGx motif present at the self-cleavage site is absent in teneurin, indicating that the autoproteolytic activity has been lost (Figure 2C). Accordingly, there is no evidence of proteolytic cleavage in the crystal structure of chicken Ten2.

The C-terminal regions of TcC proteins contain a highly variable toxin domain encoding a variety of functions, including the ADP-ribosylation of actin and Rho proteins, and a predicted nucleic acid deaminase (Lang et al., 2010, 2011; Marshall et al., 2012). The equivalent region in teneurin instead contains a conserved linker domain that exits the hollow shell through a gap in its side, followed by an antibiotic-binding domain (ABD) and a Tox-GHH domain which are described below.

### Bacterial Teneurin-Like Proteins

Jackson et al. (2018) identified an Rhs-repeat containing protein from *Bacillus subtilis* that shows greater similarity to teneurin than to the TcB-TcC complexes. Like teneurins, this protein is encoded by a single gene, rather than as a heterodimeric assembly. This protein contains a series of N-terminal bacterial immunoglobulin-like (Ig-like) domains in an equivalent position to the EGF repeats of teneurin. Following this is a similar set of domains to teneurin: a TTR-like domain, FN-plug, NHL β-propeller, YD-repeats, and an Rhs-repeat-associated core domain (Figure 1C). Using this sequence to search the Uniprot database reveals a number of similar proteins from diverse bacterial phyla, including Acidobacteria, Alpha, Beta, Gamma and Deltaproteobacteria, and Verrucomicrobia. While these proteins have little or no sequence similarity upstream of the TTR-like domain, they often contain a set of repeats that fold into β-sandwich domains (such as bacterial Ig-like, fibronectin-like, carboxypeptidase regulatory-like, and CARDB domains). Following these repeats, the amino acid sequence conservation increases prior to the TTR domain and continues throughout the FN-plug, β-propeller, YD-shell, and Rhs-repeat-associated core domain. In these proteins, all of the key catalytic residues involved in self-cleavage are conserved, and presumably the Rhs-repeat-associated core domain will cleave the polypeptide backbone, as is the case in TcB-TcC, leaving the C-terminal domain untethered but encapsulated inside the YD-shell. The C-terminal domain itself is highly variable, with little or no sequence conservation between species. This is in line with previous reports suggesting that YD-repeats and their associated C-terminal regions are evolutionarily decoupled in bacteria (Jackson et al., 2009). There is no indication of a linker as is found in teneurins, or that the C-terminus exits the shell. Presumably these bacterial teneurin-like proteins encapsulate their C-terminus in a manner similar to TcB-TcC complexes, rather than displaying it on the outside of the shell as is the case with eukaryotic teneurin. Bacterial proteins containing YD-repeats are widespread (Zhang et al., 2012) and are thought to be involved in toxin delivery into adjacent cells. In the case of ABC toxin complexes, this is the delivery of cytoskeleton-disrupting toxins into a eukaryotic host cell (Waterfield et al., 2001; Ffrench-Constant and Waterfield, 2006; Hurst et al., 2011), whereas other (non-ABC) YD-repeat proteins are thought to be involved in contact-dependent growth inhibition by delivering a protein toxin into other bacterial cells (Koskiniemi et al., 2013). The function of bacterial teneurin-like proteins is currently unknown, but is likely to involve the delivery of the variable C-terminal domain into other cells, possibly to inhibit the growth of competing microorganisms, or during pathogenesis.

## Teneurin Domains Downstream of the Scaffold

Approximately 500 amino acids link the vertebrate teneurin scaffold region to its single-span transmembrane helix. This region comprises a membrane-proximal region of ∼180 amino acids, eight epidermal growth factor-like (EGF) repeats, a short cysteine-rich motif and a recently identified transthyretin-like (TTR) domain, which is flexibly linked to the central scaffold and packs against the FN-plug domain in the high resolution crystal structure of chicken Ten2, where it is stabilized in a discrete conformation by crystal lattice contacts (Jackson et al., 2018). The TTR domain is not resolved, presumably due to its flexibility, in any of the available structures determined by cryo-electron microscopy (Jackson et al., 2018; Li et al., 2018). The EGF region has not yet been structurally resolved, but bears sequence similarity to the extracellular matrix protein tenascin (Baumgartner et al., 1994; Levine et al., 1994), and has received particular attention due to its ability to dimerise teneurins (Feng et al., 2002). Two of these eight EGF repeats contain only five cysteine residues, compared to the six which would be typical in an EGF repeat. This results in the formation of intermolecular disulphide bridges that presumably form *in cis* i.e., between teneurin molecules expressed on the same cell. Early rotary shadowing electron microscopy data revealed the overall architecture of these dimers, showing pairs of globular domains (now thought to represent the teneurin scaffold and peripheral TTR, ABD and Tox-GHH), connected by thin elongated rods, presumed to be the EGF repeats (Feng et al., 2002).

Like the NHL domain, the teneurin EGF region also contains an alternatively spliced exon, which lies between the seventh and eighth EGF repeat. Sequencing of mRNA from hippocampal neurons (Berns et al., 2018) revealed three splice variants at this site. Similar to the alternatively spliced site in the NHL domain, inclusion of the alternatively spliced residues of the EGF region enables teneurin homophilic interactions *in trans* in an *in vitro* cell aggregation assay (Berns et al., 2018). Future structures of full teneurin ectodomain dimers will likely be valuable in understanding the molecular basis underlying this splicing-dependent homophilic adhesion.

Downstream of the EGF region lies a highly conserved cysteine-rich motif with the consensus sequence ExxCx(D/N)xxDx(D/E)xDxxxDCxxx(D/E)CCxxxxCxxxxxC. The teneurin cysteine-rich motif has not yet been functionally characterized, although bioinformatic analysis has revealed sequence similarity to putative metal-binding motifs in bacterial and algal proteins (Tucker et al., 2012).

## Teneurin Domains Downstream of the Scaffold

**FIGURE 3:**
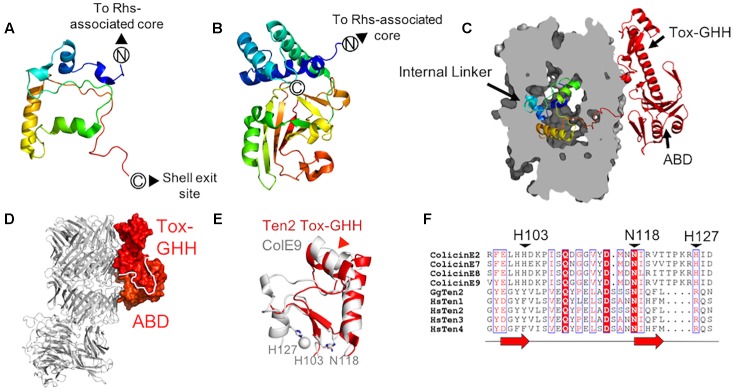
Teneurin domains downstream of the central scaffold region **(A,B)** The chicken Ten2 internal linker **(A)** as compared to the crystal structure of the cytotoxic C-terminal region of a *Y. entomophaga* C-protein (Busby et al., in preparation; PDB 6AQK) **(B)**. Both are colored from N- to C-terminus according to the rainbow. **(C)** The chicken Ten2 internal linker domain is shown as ribbons inside a clipped surface representation of the Ten2 YD-shell (gray). The ABD and Tox-GHH are shown as red ribbons. **(D)** The Ten2 ABD and Tox-GHH domain are shown in surface representation and colored as in Figure 1A. The rest of the protein is shown as gray ribbons, oriented as in Figure 1A. **(E)** Structural alignment of the Ten2 Tox-GHH domain (red ribbon) and the nuclease domain of colicin E9 (white ribbon, PDB 1BXI). Side chains of the catalytically important residues and ions in colicin E9 are shown as sticks and spheres, respectively. The position of the predicted TCAP proteolysis site in Ten2 is indicated with a red arrowhead. **(F)** Structure-based sequence alignment of the *Escherichia coli* DNase colicins, all four human teneurin paralogs and chicken Ten2. Black arrowheads indicate the catalytically important residues of the colicins. Each red arrow corresponds to one β-strand.

As predicted from previously solved structures of YD-repeat-containing proteins (Busby et al., 2013; Meusch et al., 2014), the residues immediately downstream of the Rhs-associated core (residues 2467–2592 in chicken Ten2 and 2382–2507 in murine Ten3) reside inside the shell cavity. These residues form extended loops and short helices, which pack against the Rhs-associated core domain, FN-plug and shell interior, sealing small gaps in the YD-shell. This region has been designated the “internal linker” and has no detectable structural homologs (Figure 3A). The teneurin internal linker is significantly smaller than the proteolytically cleaved C-terminal regions of TcB-TcC complex C-proteins (Figure 3B). In contrast to the teneurin internal linker, the cytotoxic C-terminal regions of bacterial C-proteins are thought to be unfolded when encapsulated within the YD-shell, and fold only upon injection into their insect cell targets (Busby et al., 2013; Meusch et al., 2014;Gatsogiannis et al., 2018).

Strikingly, unlike structures of TcB-TcC toxin complexes, where the entire C-terminal toxic domain is enclosed within the YD-shell, the teneurin C-terminal domain leaves the shell interior via a gap in the shell wall (Figure 3C). These exposed C-terminal residues (∼200 amino acids) form an intricate fold comprising two subdomains, which pack against the shell exterior (Figure 3D). This external C-terminal domain is only fully resolved in the crystal structure of chicken Ten2 and is either completely or partially disordered in both of the teneurin structures solved by single-particle electron microscopy, presumably due to conformational flexibility in this region.

Immediately downstream of the shell exit site, the crystal structure of chicken Ten2 reveals a 120-residue domain (the antibiotic-binding-like domain, ABD), which bears structural homology to a class of bacterial proteins which bind the small-molecule antibiotics bleomycin and zorbamycin (Maruyama et al., 2001; Rudolf et al., 2015). These bacterial proteins confer resistance to the antibiotics by sequestering them, preventing their activation by oxygen (Rudolf et al., 2015). The bleomycin-binding surface of the bacterial protein has been identified crystallographically (Maruyama et al., 2001). The equivalent surface in chicken Ten2 is solvent-exposed, but there are currently no known small molecule ligands for the teneurin ABD.

The ABD wraps around a long helix that leads into the most C-terminal domain of teneurin – the ToxGHH domain. The ToxGHH domain was originally identified bioinformatically and is prevalent amongst bacterial toxins, where it encodes a putative nuclease activity mediated by a HNH catalytic triad (Zhang et al., 2012). The closest structural homolog of the teneurin Tox-GHH domain (residues 2720–2795 in chicken teneurin 2) is the catalytic nuclease domain of a class of bacteriocins known as group A colicins (Figure 3E; Kleanthous et al., 1999). Structural comparison and sequence alignments of teneurin Tox-GHH domains and colicin nuclease domains reveals that teneurins lack both catalytically important histidine residues, and retain only the structurally important asparagine residue (N2790) (Figure 3F; Kleanthous et al., 1999). Recent reports have indicated that, when expressed in isolation, the teneurin C-terminal domain (chicken Ten 1 S2346-R2705 or chicken Ten2 F2407-R2802) is capable of cleaving mitochondrial circular nucleic acids *in vitro* and inducing apoptosis *in vivo*. However, in the context of an intact teneurin ectodomain, no nuclease activity was observed (Ferralli et al., 2018). Given the newly available structural data indicating that the teneurin C-terminal domain is solvent-exposed, rather than encapsulated within the YD-shell, future experiments will be necessary to determine how any potential nuclease activity is regulated.

It has previously been shown that the final ∼40 residues of the teneurin Tox-GHH domain bear sequence similarity to pre-pro-hormones and neuropeptide precursors (Qian et al., 2004). This region corresponds to the teneurin C-terminal associated peptide (TCAP), which is thought to function independently of intact teneurins to stimulate neurite outgrowth, regulate dendritic morphology and modulate anxiety behaviors in rats (Qian et al., 2004; Wang et al., 2005; Al Chawaf et al., 2007; Tan et al., 2008). As well as being transcribed independently (Chand et al., 2013), TCAPs may be generated via proteolytic cleavage from full-length teneurins (Lovejoy et al., 2006). The predicted cleavage site (based on alignment with known neuropeptides) (Lovejoy et al., 2006) is located within the second helix of the Tox-GHH domain, suggesting that the protein may unfold in this region to become accessible to proteases (Figure 3E). Recent evidence also suggests that the C-terminus of teneurin is required for binding to the adhesion-G-protein-coupled receptor latrophilin (Lphn) (Silva et al., 2011; Li et al., 2018) as removal of 390 residues from the C-terminus of human Ten2 abolished Lphn binding (Li et al., 2018). The precise nature and stoichiometry of the Lphn-Ten interaction is currently unknown.

## Evolution of Teneurin-Like Proteins

A bioinfomatic approach using a wide range of fully sequenced genomes identified teneurins in chordate genomes (e.g., mouse, chicken and zebrafish), and also in the protochordates *Ciona intestinalis* and *Branchiostoma floridae* (Tucker et al., 2012). Teneurin genes were also identified in molluscs, annelids, trematodes, nematodes and arthropods. No teneurin sequences could be identified in cnidarians, ctenophores or sponges, indicating either that they are not present in these deeper-branching eukaryotic clades, or that they have highly divergent sequences (Tucker et al., 2012). However, a single teneurin gene in the genome of the choanoflagellate *Monosiga brevicollis* was discovered and shown to have only three introns. This gene shows strong sequence similarity to some bacterial teneurin-like proteins (with strongest similarity to the teneurin-like protein from *B. subtilis*; 69% coverage, 30% identity), albeit having a divergent intracellular domain, providing evidence that the gene could have been acquired by *M. brevicollis* from its prokaryotic prey via a horizontal gene transfer event (Tucker et al., 2012), as observed for other proteins (Foerstner et al., 2008). As choanoflagellates are considered to be the closest living relatives of metazoans, the authors suggest that teneurins may have played a key role in the evolution of multicellular metazoa from their unicellular choanoflagellate ancestors. Further testing of this hypothesis awaits a careful examination of recently sequenced genomes in the light of the recently determined structures described above.

## Concluding Remarks

Recent breakthroughs in understanding the first structures of teneurin protein domains have revealed unexpected new insights into this enigmatic family of proteins. First, the structures show that the C-terminal Tox-GHH/TCAP domains are solvent-exposed and therefore will be accessible to external ligands, proteases and other extracellular factors. Second, teneurin family proteins contain a previously unknown type of fibronectin domain (FN-plug) that specifically acts to seal off the YD-shell. Third, understanding the fold of the central teneurin scaffold led to the discovery of a whole family of teneurin-like proteins in prokaryotes, suggesting evolution from an ancient and widespread fold. Future research on these bacterial proteins, comparing their structure-function relationship with teneurins, will likely reveal more unexpected aspects of teneurin biology. Last but not least, the elaborate structure of the teneurin extracellular C-terminus is unlike that of any other cell adhesion receptor, most of which consist of smaller adhesion domain repeats. The intricate nature of this fold, and the presumably large energetic cost of synthesizing and correctly folding these large proteins, suggests that teneurins may encode functions beyond the role of a canonical cell adhesion receptor that are yet to be explored.

## Author Contributions

All authors contributed to the conception and writing of the manuscript.

## Conflict of Interest Statement

The authors declare that the research was conducted in the absence of any commercial or financial relationships that could be construed as a potential conflict of interest.
